# No increase of device associated infections in German intensive care units during the start of the COVID-19 pandemic in 2020

**DOI:** 10.1186/s13756-022-01108-9

**Published:** 2022-05-07

**Authors:** Christine Geffers, Frank Schwab, Michael Behnke, Petra Gastmeier

**Affiliations:** 1 Charité – Universitätsmedizin Berlin, corporate member of Freie Universität Berlin and Humboldt-Universität zu Berlin, Institute of Hygiene and Environmental Medicine, Hindenburgdamm 27, 12203 Berlin, Germany; 2 National Reference Center for Surveillance of Nosocomial Infections, Berlin, Germany

**Keywords:** Hospital acquired infection, Bloodstream infection, Extracorporeal membrane oxygenation, COVID-19, Intensive care

## Abstract

**Background:**

The COVID-19 pandemic may have had a substantial impact on the incidence of device-associated healthcare-associated infections (HAI), in particular in intensive care units (ICU). A significant increase of HAI was reported by US hospitals when comparing incidence rates from 2019 and 2020. The objective of this study was to investigate the development of the most relevant device-associated HAI in German ICUs during the year 2020 as compared to 2019.

**Methods:**

We utilized the data of the ICU component of the German National Reference Center for Surveillance of Nosocomial Infections (KISS = Krankenhaus-Infektions-Surveillance-System) for the period 2019–2020. We focused on central line-associated bloodstream infections (CLABSI), catheter-associated urinary tract infections (CAUTI), ventilator-associated lower respiratory infections (VALRTI) and bloodstream infections associated with the use of Extracorporeal-Life-Support-Systems (ECLSABSI). Device use was defined as the number device days per 100 patient days; device-associated infection rates as the number of device-associated infections per 1000 device days. To compare the pooled means between the years and quarters we calculated rate ratios of device-associated infection rates with 95% confidence intervals by Poisson regression models.

**Results:**

The number of participating ICUs in the surveillance system decreased from 982 in 2019 to 921 in 2020 (6.2%). Device utilization rates increased significantly for central lines and ventilator use. VALRTI rates and CAUTI rates decreased in 2020 compared with 2019, however, no increase was shown for CLABSI or ECLSABSI. This result was also confirmed when the corresponding quarters per year were analyzed.

**Conclusions:**

The lack of an increase in device-associated healthcare associated infections (HAI) in German ICUs may be due to the lower overall incidence of COVID-19 cases in Germany in 2020 compared with US, to a very high availability of ICU beds per 100,000 inhabitants compared with many other countries, and a change in the ICU patient mix due to numerous elective procedures that were postponed during the first two waves. The primary reason seems to be that only 7% of all ICU patients in Germany in 2020 were COVID-19 patients.

## Introduction

One and a half years after the start of the COVID-19 pandemic, the first data comparing the infection rates to the pre-pandemic phase from hospitals has been published. It describes a significant increase in healthcare-associated infections (HAI) in US hospitals in 2020 [1–3]. Weiner-Lastinger et al. used data from the National Healthcare Safety Network (NHSN) from more than 3000 acute care hospitals for 2019 and 2020. Significant increases in the national standardized infection ratios (SIRs) for central-line-associated bloodstream infections (CLABSIs) (47.0%), catheter-associated urinary tract infections (CAUTIs) (18.8%), ventilator-associated events (VAEs) (44.8%), and MRSA bacteremia (33.8%) were observed in the last quarter of 2020 [1]. Baker et al. described an even higher increase. Using data from 148 acute care hospitals in 17 US states, they calculated 60% more CLABSIs, 43% more CAUTI, and 44% more cases of MRSA bacteremia than expected over the seven month period from 1 March 2020 to 30 September 2020 [3]. The HAI Progress Report of the NHSN also included data on the situation in intensive care units (ICUs), with a 50% increase in CLABSI followed by a 35% increase in ventilator-associated events, and a 10% increase in CAUTI between 2019 and 2020 [2].

Therefore, the objective of this study was to investigate the development of the most relevant device-associated HAI in German ICUs during the year 2020 as compared to 2019. Because many COVID-19 patients require therapy with extracorporeal life support systems (ECLS), such as ECMO (extracorporeal membrane oxygenation), and because the ICU component of the German national surveillance system also performs surveillance of ECLS-associated bloodstream infections (ECLSABSI), special attention was given to this infection type.

## Methods

We used data from the ICU component of the national surveillance system for healthcare associated infections in Germany (KISS = Krankenhaus-Infektions-Surveillance-System) for the period 2019–2020. The KISS method of surveillance in ICUs is based on the NHSN method [4]. However, the definitions for CLABSI and CAUTI have been slightly modified, and we focused on ventilator-associated lower respiratory tract infections (VALRTI) rather than VAEs. In addition, we performed surveillance for primary bloodstream infections associated with the use of extracorporeal life support systems (ECLS) by calculating ECLS utilization rates and ECLSABSI rates.

Device use was defined as the number of device days per 100 patient days. Device-associated infection rates were defined as the number of device-associated infections per 1000 device days. We calculated the yearly metrics for each ICU, including their distributions, as a median and interquartile range (IQR) and the pooled means per year and quarter. The primary analysis was a comparison of the metrics between the years 2019 and 2020. Differences were tested using the Chi-Square test or the Wilcoxon rank-sum test. A* p* value of less than 0.05 was considered statistically significant. To compare the pooled means between the years and quarters, we calculated the rate ratios of device-associated infection rates with 95% confidence intervals using Poisson regression models with the outcome device-associated infections and logarithmized device days as the offset parameter. All analyses were exploratory in nature and performed with SPSS (version 25) and SAS (version 9.4).

## Results

Table 1 describes the characteristics of the participating ICUs. There was a decrease from 982 ICUs in 2019 to 921 ICUs in 2020 (6.2%).

**Table 1:** Characteristics of ICUs participating in ICU-KISS (Germany) in the years 2019 and 2020.

Parameter	Category/description	Year		*P* value
		2019	2020	
		N(%)	N(%)	
ICUs	Total	982 (100%)	921 (100%)	
Type of ICU	Interdisciplinary in a hospital < 400 beds	382 (38.9%)	352 (38.2%)	0.996
	Interdisciplinary in a hospital ≥ 400 beds	190 (19.3%)	178 (19.3%)	
	Medical	119 (12.1%)	108 (11.7%)	
	Surgical	124 (12.6%)	112 (12.2%)	
	Neurosurgical	17 (1.7%)	19 (2.1%)	
	Cardiosurgical	29 (3%)	31 (3.4%)	
	Neurologic	34 (3.5%)	35 (3.8%)	
	Pediatric	24 (2.4%)	21 (2.3%)	
	Other than above	63 (6.4%)	65 (7.1%)	
Size of ICU (beds)	Sum	13,941	12,652	
	Median (IQR)	12 (10–16)	12 (10–16)	0.921
Size of hospital (beds)	Sum	536,549	517,836	
	Median (IQR)	415 (248–684)	420 (245–735)	0.540

ICU, intensive care unit; IQR, interquartile range

The mean number of ICU patients in the surveillance system declined by 13.8% in 2020 in comparison to 2019 (Table 2). Length of stay increased slightly from 3.8 to 3.9 days. Device utilization rates increased significantly for central line and ventilator use. VALRTI rates and CAUTI rates decreased in 2020 compared with 2019; however no statistically significant increase was identified for CLABSI and ECLSABSI.

**Table 2:** Comparison of device utilization rates and device-associated infection rates in intensive care units in the German infection surveillance system (ICU-KISS) between the years 2019 and 2020

Parameter	Description	Year 2019	Year 2020	*P* value*
		N = 982 ICUs	N = 921 ICUs	
ICU with HAI surveillance				
Patients included per year	Sum	863,999	696,085	
	Median (IQR)	814.5 (527–1114)	689 (427–983)	** < 0.001**
Patient days	Sum	3,296,045	2,731,371	
	Median (IQR)	3124 (2121–4211)	2679 (1819–3749)	** < 0.001**
LOS (days)	Pooled mean Median (IQR)	3.8 3.8 (2.9–5.0)	3.9 3.9 (3.0–5.2)	0.125
CLABSI surveillance				
Central line use (central line days per 100 patient days)	Pooled mean Median (IQR)	64.1 63.6 (49.1–77.5)	66.1 66.2 (52.4–79.5)	**0.012**
CLABSI	No	2372	2088	
CLABSI rate (CLABSI per 1000 central line days	Median (IQR)	0.7 (0–1.59)	0.64 (0–1.63)	0.263
	Pooled mean (95%CI)	1.12 (1.08–1.17)	1.16 (1.11–1.21)	
	RR 2020 versus 2019 (95%CI)		1.03 (0.97–1.09)	0.340
VALRTI surveillance				
Ventilator use (ventilator days per 100 patient days)	Pooled mean Median (IQR)	37.0 32.5 (22.4–45.9)	37.8 35.1 (24.4–46.4)	**0.043**
VALRTI	No	4942	3731	
VALRTI rate (VALTRI per 100 ventilator days)	Median (IQR)	2.95 (0.76–5.68)	2.02 (0–4.91)	** < 0.001**
	Pooled mean (95%CI)	4.09 (3.98–4.21)	3.63 (3.52–3.75)	
	RR 2020 versus 2019 (95%CI)		0.89 (0.85–0.93)	** < 0.001**
CAUTI surveillance				
Catheter use (catheter days per 100 patient days)	Pooled mean Median (IQR)	81.6 84.2 (74.8–90.9)	82.1 84.6 (76.0–91.5)	0.259
CAUTI	No	3286	2589	
CAUTI rate (CAUTI per 1000 catheter days)	Median (IQR)	0.61 (0–1.63)	0.49 (0–1.47)	**0.008**
	Pooled mean (95%CI)	1.23 (1.18–1.27)	1.16 (1.11–1.20)	
	RR 2020 versus 2019 (95%CI)		0.94 (0.90–0.99)	**0.028**

		N = 68 ICUs	N = 65 ICUs	*P* value
ECLSABSI surveillance				
Patients		52,120	39,153	
Patient days		213,602	175,318	
ECLS-days		13,287	12,175	
Use of extracorporal life support systems (ECLS)				
ECLS-days per 100 patient days	Pooled mean Median (IQR)	6.2 3.8 (1.8–7.8)	6.9 3.4 (2.25–7.8)	0.918
ECLSABSI	No	17	16	
ECLSABSI rate (ECLSABSI per 1000 ECLS days)	Median (IQR)	0 (0–0)	0 (0–0)	0.994
	Pooled mean (95%CI)	1.28 (0.75–2.05)	1.31 (0.75–2.13)	
	RR 2020 versus 2019 (95%CI)		1.03 (0.52–2.03)	0.939

Bold values indicate the statistically significant =* p* < 0.05

ICU, intensive care unit; LOS, length of stay; No, number; Med, median; IQR, interquartile range; 95%CI, 95% confidence interval; RR, rate ratio; CLABSI, Central line-associated bloodstream infection; CL, central line (central venous catheter);VALRTI, ventilator associated lower respiratory tract infection; CAUTI, Catheter associated urinary tract infection; ECLSABSI, Extracorporeal-Life-Support-Systems associated bloodstream infection

*p values, calculated by Chi-square test or Wilcoxon rank-sum test based on the yearly data of ICUs or by Poisson regression; Rate ratios (RR) are calculated by Poisson regression

Only 68 ICUs provided data on the use of ECLS in 2019 (6.9%); 65 ICUs did in 2020 (7.0%). The mean ECLS utilization rate increased from 6.2 to 6.9 per 100 patient days. The absolute number of ECLSABSI was low, with 17 and 16 infections respectively. The majority of ICUs with ECLS days had no ECLSABSI. The pooled mean was 1.28 in 2019 and 1.31 per 1000 ECLS days in 2020.

**Fig. 1:**
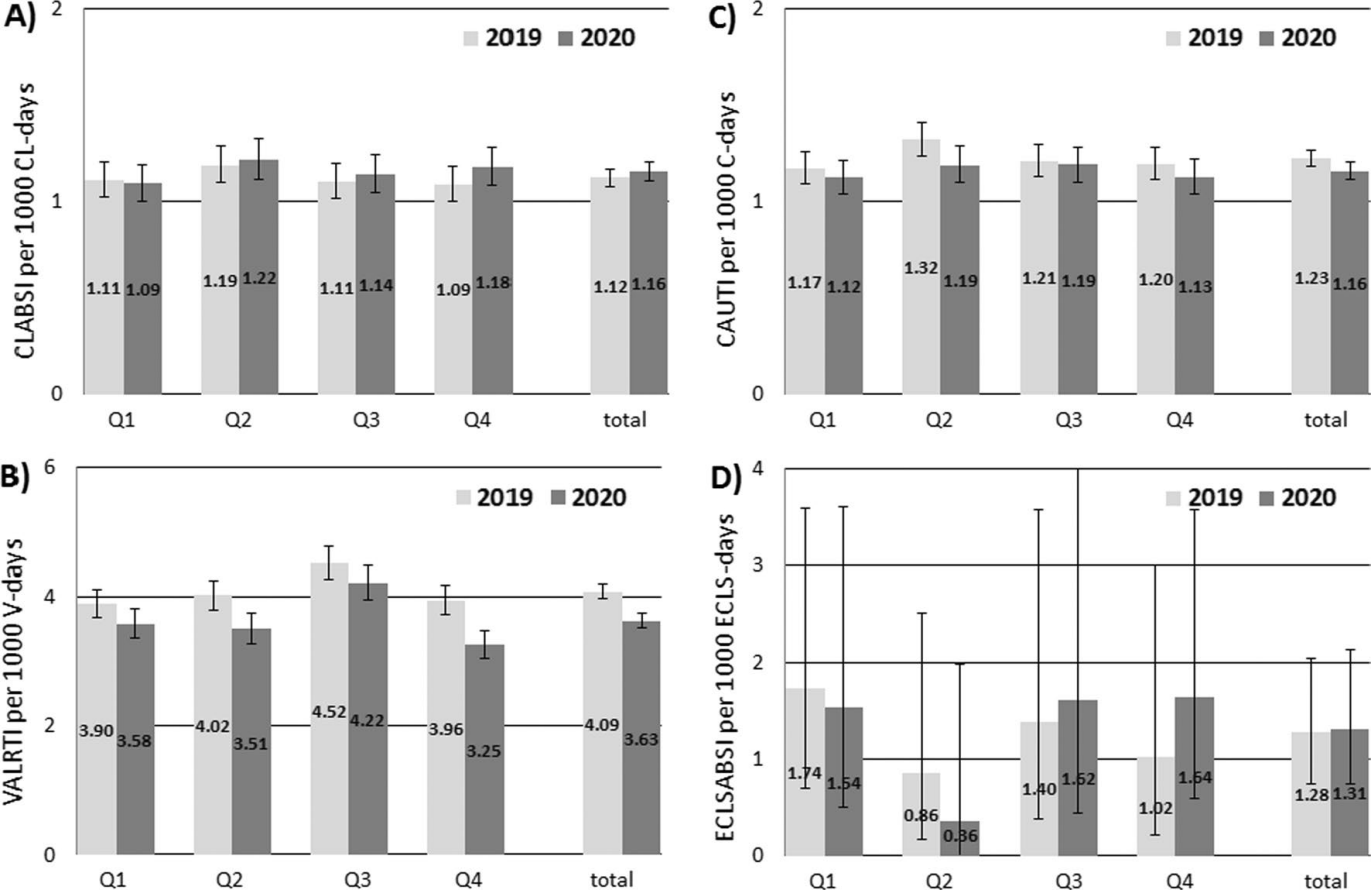
**A**–**D** Pooled mean device associated infection rates with 95% confidence intervals in the year 2019 and 2020 in total and by quarter, ICU-KISS (Germany)

Figure 1 also shows pooled mean device-associated infection rates by quarter in the years 2019 and 2020 with 95% confidence intervals.

Table 3 shows the difference between the two years with 95% confidence intervals for the four quarters of 2020. According to this analysis, the decrease in VALRTI was significant in the first, second, and fourth quarters of 2020. The decrease in CAUTI was significant only in the second quarter of 2020. The changes in CLABSI were rather small and did not achieve a level of significance in any quarter. The ECLSABSI rates increased substantially during the 4th quarter, but this increase was not significant. The median ECLSABSI rates were about twice as high as the median CLABSI rates (0.64 vs 1.31 per 1000 device days) in 2020.

**Table 3:** Change in device-associated infection rates in 2020 compared to 2019, ICU KISS (Germany)

Type of infection	2020 (total)	2020 Q1	2020 Q2	2020 Q3	2020 Q4
CLABSI	1.03 95% CI 0.97–1.09 *p* = 0.340	0.97 95% CI 0.877–1.108 *p* = 0.8113	1.03 95% CI 0.912–1.152 *p* = 0.6768	1.04 95% CI 0.92–1.17 *p* = 0.568	1.08 95% CI 0.96–1.22 *p* = 0.197
VALRTI	0.89 95% CI 0.85–0.93 *p* < 0.001	0.92 95% CI 0.85–0.998 *p* = 0.044	0.87 95% CI 0.80–0.95 *p* = 0.002	0.93 95% CI 0.86–1.01 *p* = 0.100	0.82 95% CI 0.75–0.90 *p* < 0.001
CAUTI	0.94 95% CI 0.90–0.99 *p* = 0.028	0.96 95% CI 0.87–1.06 *p* = 0.413	0.90 95% CI 0.81–0.998 *p* = 0.045	0.98 95% CI 0.89–1.09 *p* = 0.7304	0.94 95% CI 0.85–1.05 *p* = 0.2754
ECLSABSI	1.03 95% CI 0.52–2.03 *p* = 0.939	0.89 95% CI 0.28–2.79 *p* = 0.834	0.41 95% CI 0.04–3.97 *p* = 0.444	1.16 95% CI 0.29–4.64 *p* = 0.833	1.60 95% CI 0.40–6.40 *p* = 0.506

CLABSI, Central line associated bloodstream infection; VALRTI, Ventilator associated lower respiratory tract infection; CAUTI, Catheter associated urinary tract infection; ECLSABSI, Extracorporeal-Life-Support-Systems associated bloodstream infection; total, January-December; Q1, January-March; Q2, April-June; Q3, July–September; Q4, October-December; Changes are calculated as rate ratios of device associated infections using Poisson regression models with the outcome number of infection and logarithmized device days as offset parameter

## Discussion

In contrast to the data from US hospitals, we did not observe an increase in the most relevant device associated HAI in German ICUs. Other than in the article by Weiner-Lastinger et al. we concentrated only on ICUs [1]. However, device utilization rates are usually highest in ICUs and the pandemic’s greatest impact is to be expected in ICU. Therefore, a focus on ICUs may be useful for an analysis of the development in Germany.

It is plausible that HAI rates could increase during a pandemic. The care of COVID-19 patients is associated with a more frequent use of personal protective equipment and a disruption of routine infection control practices. In addition, many hospitalized COVID-19 patients receive corticosteroid therapy, which may increase the risk of developing super-infections [5]. The risk for severe COVID-19 infection increased for patients with comorbidities. In addition, many elective procedures were postponed during the first two COVID-19 waves. The influence of such decisions on the overall ICU patient mix is difficult to calculate but therefore, we assume that many patients had comorbidities and other risk factors for device-associated HAI.

Overcrowding and/or understaffing was observed in many hospitals—both well-known risk factors for the development of HAI [6, 7]. The stable situation with regard to device-associated infections in German ICUs may be due to the fact that there was no overcrowding in most ICUs. This can be attributed to the following primary factors:

- The overall COVID-19 incidence was lower in Germany than the US in 2020
- The much higher availability of ICU beds per 100 000 inhabitants in Germany than in the US and many other countries, and
- A change in the ICU patient mix due to the large number of elective procedures that were postponed during the first two waves.

According to WHO data, in the US the incidence of COVID-19 infections was 13,416 per 100,000 inhabitants in 2020 compared to 5259 per 100,000 inhabitants in Germany [8]. As the German Federal Statistical Office (Destatis; https://www.destatis.de) reports, Germany had 33.9 intensive care beds per 100,000 inhabitants. In the United States, the ratio was 25.8 intensive care beds per 100,000 inhabitants, in Spain the ratio was 9.7 and in Italy 8.6 intensive care beds per 100,000 inhabitants [9]. Thus, we had a relatively good ratio the total capacity of ICU beds to the number of COVID-19 ICU patients in 2020.

A study from Switzerland showed a positive correlation of ICU BSI with the ICU occupancy rate due to COVID patients [14]. Correlation was also shown in a hospital group in London between the proportion of COVID patients in ICUs and BSI [13]. According to statistics from the German Interdisciplinary Association for Intensive Care and Emergency Medicine (DIVI), beginning on 24.04.2020 the overall percentage of COVID-19 patients among all ICU patients in Germany was 7% until the end of 2020 with a peak in the last week of the year when this percentage reached 26% [10]. Therefore, the system in Germany was less affected by overcrowding.

The decrease of VALRTI that was observed may be explained by the fact that it is difficult to diagnose a nosocomial lower respiratory tract infection as new in patients who were admitted with a LRTI. The decrease in CAUTI may be due to the previously mentioned change in the patient mix and a higher rate of antibiotic use during the two COVID-19 waves [11], 12. The unchanged CLABSI rate is the most interesting finding. This stands not only in contrast to the data from US hospitals but also to data from English and Swiss hospitals [13, 14].

To our knowledge, no other national surveillance system is also surveying BSI associated with ECLS use, only single center studies of the incidence of ECLSABSI are available [15, 16]. We were surprised that the use of ECLS devices did not increase significantly during the pandemic or that the ECLS-associated infection rates did not increase either. This could be explained by the fact that the German Interdisciplinary Association for Intensive Care and Emergency Medicine (DIVI) created a network of ICUs at the beginning of the pandemic which classified clearly which ICUs were supposed to care for COVID-19 patients. Only those with the most experience in this area were selected. In addition, this network was also very useful for the regional management of COVID-19 patients and for avoiding overcrowding.

Our study has some limitations:

 First, we concentrated on ICU patients only. However, this is probably the patient group in the hospital with the highest risk of device-associated infections and which suffered the greatest potential impact from the pandemic.

 Secondly, we do not have patient-related data. We are therefore unable to make any statements on a possible shift in risk due to a change in patient mix.

 Third, the percentage of participating ICUs decreased by 6.2% between 2019 and 2020, and the number of patients included in the study decreased by 13.8%. This maybe means that some larger ICUs were unable to participate in the ICU component of KISS probably due to the surveillance staff’s generally higher workload. These 6% of the ICUs could possibly have higher infection rates, especially if staff shortages were indeed the reason that the data were not provided. However, a relevant influence on the results of this study is not to be expected, especially the 13,8% less patients could also be the result of lower treatment numbers in Germany in 2020.

 Fourth, it may have been difficult for infection control teams to maintain the same degree of accuracy when diagnosing HAI on ICUs where there was a relatively large number of COVID-19 patients. However, the vigilance in connection with HAI may have even increased in other ICUs during the pandemic.

Altogether, the study underscores the need for a high standard of baseline infection control measures as well as the fact that this should remain unchanged during a pandemic.

The results from Germany in this study complement previous literature from other countries with lower numbers of ICU beds per 100,000 inhabitants. This study underscores the need to consider the structural conditions when interpreting studies on the effect of the pandemic.

Despite the different findings by the NHSN and KISS, the data demonstrates the value of these national surveillance systems for analyzing a situation and drawing conclusions for infection management in the future.

## Conclusions

The lack of an increase in device-associated healthcare associated infections (HAI) in German ICUs during the pandemic year 2020 may be due to the lower overall incidence of COVID-19 cases compared with US, to a very high availability of ICU beds per 100,000 inhabitants compared with many other countries accompanied by a change in the ICU patient mix due to numerous elective procedures that were postponed during the first two waves.

## Data Availability

The datasets used and analyzed in the context of this survey are available from the corresponding author upon reasonable request.

## Abbreviations

CAUTI: Catheter-associated urinary tract infections
CLABSI: Central line-associated bloodstream infections
COVID-19: Coronavirus Disease 2019
DIVI: German Interdisciplinary Association for Intensive Care and Emergency Medicine
ECLSABSI: Extracorporeal-Life-Support-Systems-associated bloodstream infections
ECMO: Extracorporeal membrane oxygenation
HAI: Healthcare-associated infections
ICU: Intensive care units
IQR: Interquartile range
KISS: Krankenhaus-Infektions-Surveillance-System
LRTI: Lower respiratory tract infections
MRSA: Methicillin-resistant *Staphylococcus aureus*
NHSN: National Healthcare Safety Network
SIRs: Standardized infection ratios
SPSS: Statistical Package für Social Sciences
VAE: Ventilator-associated events
VALRTI: Ventilator-associated lower respiratory infections

## References

[1] Weiner-Lastinger L, Pattabiraman V, Konnor R, Patel P, Wong E, Xu S, et al. The impact of coronavirus disease 2019 (COVID-19) on healthcare-associated infections in 2020. A summary of data reported to the National Healthcare Safety Network. Infect Control Hosp Epidemiol. 2021. 10.1017/ice.2021.362 34473013

[2] anonym. Current HAI Progress Report_2020 National and State Healthcare-Associated Infections Progess Report. https://www.cdcgov/hai/data/portal/progress-reporthtml. 2021.

[3] Baker M, Sands K, Huang S, Kleinamn K, Septimus E, Varma N, et al. The Impact of Coronavirus Disease 2019 (COVID-19) on healthcare associated infections. Clin Infect Dis. 2021. 10.1093/cid/ciab688 PMC8385925 34370014

[4] Emori TG, Culver DH, Horan TC, Jarvis W, White J, Olson D (1991). National Nosocomial Infection Surveillance System (NNIS): description of surveillance methodology. Am J Infect Control.

[5] Rothe K, Lahmer T, Rasch S, Schneider J, Spinner C, Wallnöfer F (2021). Dexamethasone therapy and rates of secondary pulmonary and bloodstream infections in critically ill COVID-19 patients. Multidiscip Respir Med.

[6] Lacotte Y, Ardal C, Ploy M-C (2020). Infection prevention and control research priorities: What do we need to combat healthcare-associated infections and antimicrobial resistance? Results of a narrative liteture review and survey analysis. Antimicrob Resist Infect Control..

[7] Houghton C, Meskell P, Delaney H, Smalle M, Glenton C, Booth A (2020). Barriers and facilitators to healthcare workers' adherence with infection prevention and control (IPC) guidelines for respiratory infectious diseases: a rapid qualitative evidence synthesis. Cochrane Database Syst Rev..

[8] anonym. https://www.covid19whoint. 2021. Access 15 Oct 2021.

[9] Anonym. High hospital bed density in Germany comapred with other contries. Statistisches Bundesamt https://www.destatisde/EN/Press/2020/04/PE_231htm. 2020.

[10] anonym. https://www.diviexchangeblobcorewindowsnet/%24web/zeitreihe-bundeslaendercsv. 2021.

[11] Rothe K, Feihl S, Schneider J, Wallnöfer F, Wurst M, Lukas M (2021). Rates of bacterial co-infections and antimicrobial use in COVID-19 patients: a retrospective cohort study in the light of antibiotic stewardship. Eur J Clin Microbiol Infect Dis.

[12] Stevens R, Jensen K, Kooda K, Mara K, O`Horo J, Shah A. A retrospective antibiotic prescribing assessment and examination of potential antibiotic stewardship targets in patients with COVID-19. J Antimicrob Resistance. 2021. 10.1093/jacamr/dlab170 PMC8569441 34755114

[13] Zhu N, Rawson T, Mookerjee S, Price J, Davies F, Otter J (2021). Changing patterns of bloodstream infections in the community and acute care across two COVID-19 epidemic waves: a retrospective analysis using data linkage. Clin Infect Dis..

[14] Damonti L, Kronenberg A, Marschall J, Jent P, Sommerstein R, De Kraker M (2021). The effect of the COVID-19 pandemic on the epidemiology of positive blood cultures in Swiss intensive care units: a nationwide surveillance study. Crit Care.

[15] Grasselli G, Scaravilli V, Di Bella S, Biffi S, Bombino M, Patroniti N (2017). Nosocomial infections during extracorporeal membrane oxygenation: incidence, etiology, and impact on patients' outcome. Crit Care Med.

[16] Qunitana M, Mazzeffi M, Galvagno S, Herrera D, Boyajian G, Hays N (2021). A retrospective study of infection in patinets requiring extracorporeal membrane oxygenation support. Ann Thorac Surg.

